# Cholestane-3β,5α,6β-triol induces cancer cell death by activating GSDME-mediated pyroptosis

**DOI:** 10.3389/fphar.2025.1667156

**Published:** 2025-10-24

**Authors:** Jiaxi Chen, Yuan He, Min Zhao, Zihan Liu, Zixin Su, Chuanzhou Li, Chen Yang, Jieping Zhang, Shuichun Mao, Hua Han, Zhenyu Cai, Wen Zhang

**Affiliations:** ^1^ School of Medicine, Tongji University, Shanghai, China; ^2^ College of Oceanography and Ecological Science, Shanghai Ocean University, Shanghai, China; ^3^ School of Pharmacy, Nanchang University, Nanchang, China; ^4^ Ningbo Institute of Marine Medicine, Peking University, Ningbo, China

**Keywords:** cholestane-3β,5α,6β-triol, pyroptosis, GSDME, caspase 3, inflammation

## Abstract

**Background:**

Trihydroxysterols and their analogues accumulate in several pathologies, including neurodegenerative diseases, cancers, and atherosclerosis. Cholestane-3β,5α,6β-triol (CT), recognized as an apoptosis-inducing agent, also exhibits pro-inflammatory effects. Nevertheless, the mechanisms underlying CT-induced cytotoxicity and inflammation remain incompletely characterized.

**Methods:**

RNA-sequencing (RNA-seq) analysis indicated CT can stimulate pro-inflammatory cytokine expression. We then employed multiple cell death inhibitors to confirm the predominant form of CT-induced cell death. Using combined chemical inhibition and genetic editing approaches, we established the relationship between caspase 3 activation, CT-mediated gasdermin E (GSDME) cleavage, and subsequent cell death.

**Results:**

CT promotes the expression of multiple pro-inflammatory cytokines. Among inflammatory cell death effector proteins, GSDME was exclusively highly expressed in our cell model. Notably, CT-induced cytotoxicity was abolished by either pharmacological GSDME inhibition or genetic knockdown of GSDME expression. This GSDME-dependent cell death pathway was consistently observed across multiple cell lines. Furthermore, caspase 3 silencing mitigated CT-induced GSDME cleavage, thereby enhancing cell viability.

**Conclusion:**

CT specifically triggered caspase 3-dependent GSDME cleavage, resulting in pyroptosis as the predominant form of CT-induced cell death. This study establishes a direct mechanistic link between CT and inflammatory cell death execution and provides insight into the contribution of trihydroxysterols to inflammatory pathogenesis.

## Introduction

Autoxidized cholesterols in mammals are found to accumulate in numerous inflammatory diseases, such as neurodegenerative diseases, cancers, and atherosclerosis (Sottero et al., 2019). Cholestane-3β,5α,6β-triol (CT) is a known autoxidation product of cholesterol in mammals and is found to accumulate in tumor tissues (Jusakul et al., 2012; Voisin et al., 2017; Kloudova-Spalenkova et al., 2020).

Previous research reported that CT induces cyclooxygenase-2 (COX-2) expression via phosphoinositide 3-kinase (PI3K)-protein kinase B (Akt)-endothelial nitric oxide synthase (eNOS), p38MAPK, and NF-κB pathways, which subsequently contribute to the inflammation process (Liao et al., 2009). CT has been shown to stimulate the production of interleukin 8 (IL-8) in isolated human peripheral blood monocytes (Liu et al., 1997). However, the regulatory mechanism of CT on inflammation remains unclear.

CT exhibits cytotoxic effects on various cell types by inducing apoptosis and necroptosis (Liu et al., 2004; Liu et al., 2005; Carvalho et al., 2010; Levy et al., 2018; Attanzio et al., 2019). Our previous findings revealed that although CT does induce some apoptotic cell death, a significant proportion of cell death results from cell membrane integrity disruption (Chen et al., 2024). This observation was in line with reported data that CT-treated prostate cancer cells show no significant change in the proportion of sub-G1 cells (Lin et al., 2013). Also, no early apoptotic cells are observed in MMK-1 cells treated with CT (Jusakul et al., 2012), and CT only causes significant lactate dehydrogenase (LDH) release from ECV-304 cells, without the presence of early apoptotic cells (Wu and Huang, 2006; Liu et al., 2011). Nevertheless, existing research has not yet clarified the relationship between the various causes of death associated with CT and inflammation regulation.

Pyroptosis is an inflammatory cell death characterized by the formation of pores in the plasma membrane. The gasdermins (GSDMs) protein family serves as the executors of pyroptosis, resulting in membrane perforation, cell swelling and rupture (Jia et al., 2023). Except for Pejvakin (PJVK), other GSDMs contain a C-terminal repressor domain and an N-terminal pore-forming domain (Liu et al., 2021), which can interact with acidic phospholipids in the inner leaflet of cell membranes to form pores (Yu et al., 2021). In 2023, it was reported that 7-ketocholesterol (7-KC), another common autoxidized cholesterol, caused both necrosis and pyroptosis via specific cleavage of GSDME instead of GSDMD in retinal pigment epithelium cells. This was the first time to report the association between autoxidized cholesterol and pyroptosis effector (Pariente et al., 2023). However, a direct mechanistic connection between CT and inflammatory cell death remains unestablished.

The current study aimed to investigate the cytotoxicity of CT in cancer cells and elucidate the signaling mechanisms of CT in inflammation regulation, providing a theoretical basis for understanding how CT promotes the progression of diseases by exacerbating inflammatory processes.

## Materials and methods

### Reagents and antibodies

CT (Avanti Polar Lipids, Inc., Birmingham, United States) was dissolved in ethanol and stored at −20 °C. Necrostatin-1 stable (Nec-1s), ferrostatin-1 (Fer-1), liproxstatin-1 (Lip-1), Deferiprone (DFP), cholesterol and staurosporine (STS) were acquired from Selleck (Houston, United States). Necroptosis Inducer Kit (with TSZ), cisplatin (CDDP), Z-VAD-fmk, and puromycin dihydrochloride were purchased from Beyotime (Shanghai, China). Disulfiram (TETD) and 2-bromohexadecanoic acid (2-BP) were purchased from MedChemExpress (Monmouth Junction, United States). Phospho-mixed lineage kinase domain-like pseudokinase (MLKL) (Ser358) (E7G7P) Rabbit mAb, GSDMD (E9S1X) Rabbit mAb, cleaved GSDMD (Asp275) (E7H9G) Rabbit mAb, caspase 3 antibody (CAT#9662S), cleaved caspase 3 (c-caspase 3) (Asp175) antibody (CAT#9661T), β-actin rabbit mAb (CAT#4970) were purchased from Cell Signaling Technology (Beverly, United States). GAPDH antibody (CAT#10494-1-AP) was purchased from proteintech (IL, United States). Recombinant anti-GSDMA antibody (CAT#ab230768), recombinant anti-DFNA5/GSDME antibody (CAT#ab215191), anti-NOD-like receptor protein 3 (NLRP3) antibody (CAT#ab263899) and anti-IL-1β antibody (CAT#ab254360) were purchased from Abcam (Cambridge, United Kingdom). GSDMC Rabbit pAb (CAT#A14550) was purchased from ABclonal (Massachusetts, US). Goat anti-rabbit IgG, HRP-linked antibody (CAT#31460) was purchased from Invitrogen (Carlsbad, United States). GSDMB rabbit pAb (CAT#abs111654) was purchased from Absin Bioscience (Shanghai, China). IL-18 Rabbit mAb (CAT#R24693) was purchased from Zen-bioscience (Chengdu, China). Goat anti-mouse IgG, HRP-linked antibody (CAT#A0216) was purchased from Beyotime (Shanghai, China).

### Cell culture

A549, HepG2, MCF-7, HeLa, HT29, HEK293T, SH-SY5Y, N2a, and BV2 were cultured in Dulbecco’s Modified Eagle Medium (Procell, Wuhan, China), supplemented with 10% FBS (Viva Cell Biotechnology, Denzlingen, Germany) and 1% penicillin-streptomycin (Gibco, Carlsbad, United States). The cultures were incubated at 37 °C with 5% CO_2_.

### Cytotoxicity assay

Cells were seeded on a 96-well plate at a density of 8000 cells per well and incubated overnight. Inhibitors were pre-treated for 1 h before incubating cells with CT for 24 h. Cell viability was assessed by using the Cell Counting Kit-8 (CCK-8) (DojinDo, Nanjing, China). The absorbance was measured at 450 nm with a microplate absorbance reader (Infinite F50, TECAN, Männedorf, Switzerland) after incubation at 37 °C for 1 h.

### Total LDH release assay

Cell integrity was assessed by quantifying LDH released into the culture media upon plasma-membrane disruption. The assay was performed using the LDH Cytotoxicity Assay Kit (Beyotime, Shanghai, China) following the manufacturer’s instructions. The absorbance was measured at 492 nm with 620 nm reference absorbance. LDH Release (% of control) = Sample Absorbance/Control Absorbance × 100%.

### Apoptosis assay by flow cytometry

Flow cytometry was performed with annexin V-FITC/PI Cell Apoptosis Detection Kit (Beyotime, Shanghai, China) to detect apoptosis. Cells were seeded on a 24-well plate at a density of 70,000 cells per well. After treatment with CT, cells were collected with trypsin (Gibco, Carlsbad, United States), centrifuged at 300 *g* at 4 °C for 5 min, and washed twice with cold PBS. Cells were resuspended in 195 µL cold binding buffer. Then, 5 µL annexin V-FITC and 10 µL PI were added to cell suspensions and incubated in the dark at room temperature for 10 min. The cells were analyzed by flow cytometry (Becton Dickinson, Franklin Lakes, United States).

### Lipid peroxidation measurement

The level of lipid reactive oxygen species (ROS) in cells treated with CT was measured by using BODIPY™ 581/591 C11 (Invitrogen, Carlsbad, United States). After treatment with CT, cells were collected with trypsin, and then washed twice with cold PBS. BODIPY™ 581/591 C11 dye was prepared with HBSS (Procell, Wuhan, China) to 5 μM, and cells were resuspended in 200 μL/tube, and then incubated at 37 °C in the dark for 30 min. Shaking the tubes during incubation. After incubation, cells were measured immediately with the flow cytometer. The results were calculated as MFI_exp_/MFI_NC_ × 100%.

### RNA-sequencing (RNA-seq) assay

For RNA-seq analysis, A549 cells were collected using TRIzol reagent (Invitrogen, Carlsbad, United States) following a 24 h treatment with different concentrations of CT. Subsequently, all the samples were sent to BGI Corporation (Shenzhen, China) for further RNA-seq detection and analysis via MGISEQ-2000RS sequencer. Bioinformatics Workflow, including data filtering, mapping transcript prediction, differential gene expression analysis, and GO and Pathway analysis, was performed on the Dr. Tom network platform of BGI (http://report.bgi.com, accessed on 14/3/2023). Detailed information is available upon request. Overall, the analysis yielded an average of 1.18 G clean reads, with an average genome mapping rate of 93.27%. A total of 17,814 genes were identified in this investigation.

### Western blot (WB) assay

Cell pellets were lysed in RIPA buffer (Beyotime, Shanghai, China) containing 1× general protease and phosphatase inhibitor cocktail (Absin Bioscience, Shanghai, China). Total protein concentration was measured by the BCA protein assay (Beyotime, Shanghai, China) according to the manufacturer’s instructions. An equal amount of protein was loaded on 8%–12% SDS–PAGE gels and electro-transferred to PVDF membranes. Blots were blocked in 5% skim milk (Becton Dickinson, Franklin Lakes, NJ, United States) or 5% BSA (Sigma-Aldrich; Merck KGaA, Darmstadt, Germany) in 0.05% TBST at room temperature for 2 h and then incubated with primary antibodies (1:1000) diluted in Primary Antibody Dilution Buffer (Abclonal, Woburn, United States) at 4 °C overnight. After being washed three times with 0.05% TBST, the membranes were incubated with Goat anti-rabbit IgG (1:20,000) at room temperature for 1 h. Band signals were visualized on an Amersham Imager 680 (GE Healthcare Life Sciences, Marlborough, United States) by using BeyoECL Star Kit (Beyotime, Shanghai, China). Band intensities were quantified by the ImageLab processing system. β-Actin or GAPDH was used as a loading control to normalize the detected proteins.

#### Real-time PCR (RT-PCR) assay

Total RNAs were isolated from tissues or cells using TRIzol reagent. cDNA was synthesized using PrimerScript RT Master Mix (Takara, Shiga, Japan). A real-time polymerase chain reaction was performed using the PowerUp™ SYBR™ Green Master Mix (Thermo Fisher, Massachusetts, US). Individual RT-PCR was performed using gene-specific primers, which were purchased from Tsingke Biotechnology (Beijing, China), as shown in Supplementary Table S1.

### RNA interference by lentivirus-derived shRNA

pLKO.1-puro plasmids containing shRNA targeting GSDME or caspase 3 were purchased from Tsingke Biotechnology (Beijing, China). HEK293T cells were co-transfected with pLKO.1-puro plasmid and packaging plasmids by Lipofectamine 2000 transfection reagent (Invitrogen, Carlsbad, United States). 24 and 48 h later, viral particle-containing supernatants were harvested, respectively. A549 cells were infected with the lentivirus vector with 8 μg/mL polybrene (Beyotime, Shanghai, China) for 24 h. After 24 h of culture, the medium was supplemented with 1 μg/mL puromycin dihydrochloride for 3 days. After all the cells in the blank group died, the normal medium was replaced to terminate the screening and continue to expand the culture.

### Statistics analysis

The differences between each group were analyzed using GraphPad Prism software (version 9.3.1, GraphPad Software, Boston, United States). For statistical analyses, data normality was assessed using the Shapiro-Wilk test, and homogeneity of variances was evaluated using the Brown-Forsythe test. The one-way ANOVA or two-way ANOVA was used for multiple comparisons. Data were presented as mean ± SD. Statistical significance was considered at *p* < 0.05.

## Results

### CT induces inflammatory cell death

**FIGURE 1:**
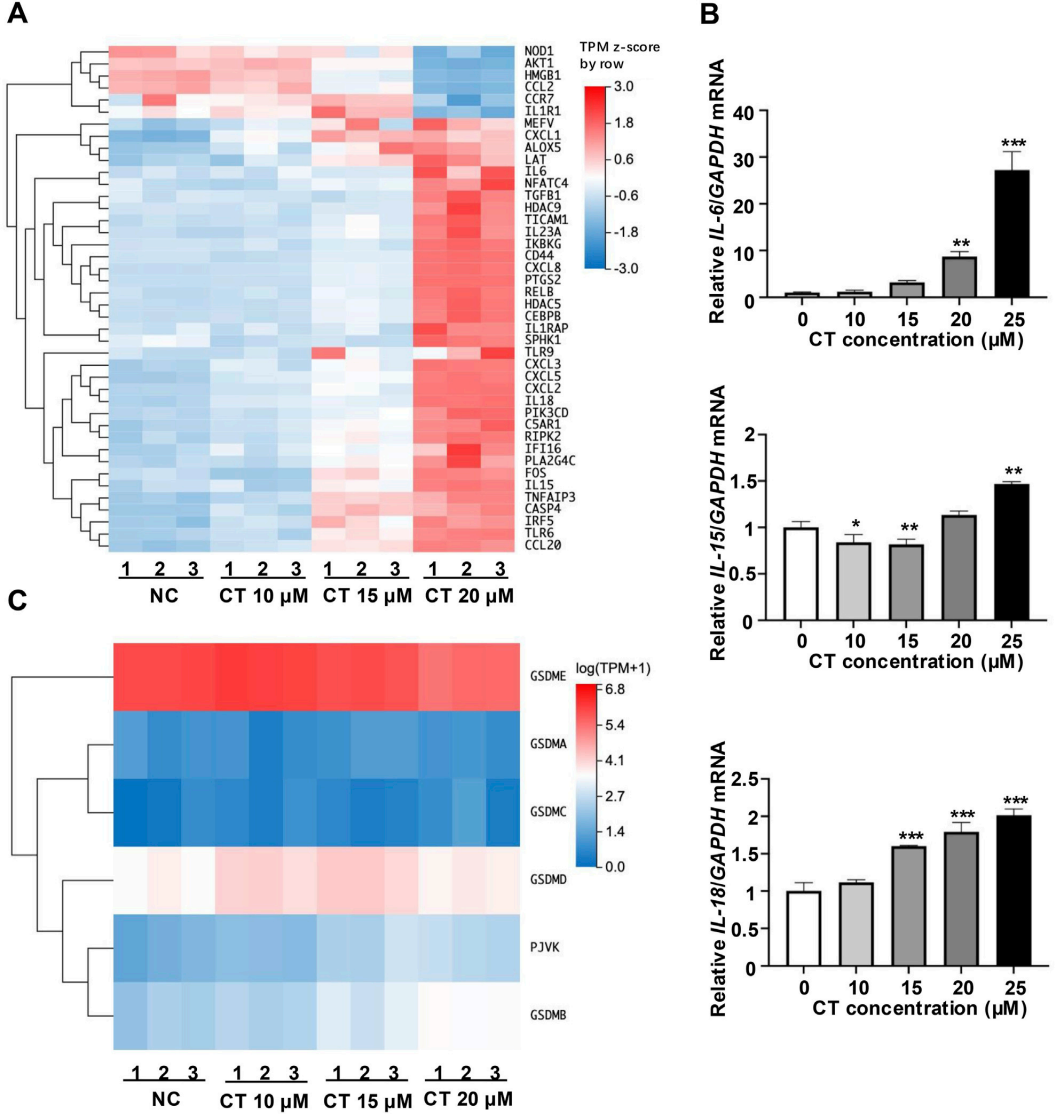
Effect of CT on the immune system in A549 cells. **(A)** Effect of CT on the transcription of genes related to inflammatory response. Cluster analysis of different expression genes (fold change ±1.5) related to inflammatory response in A549 cells treated with 10, 15 or 20 μM CT for 24 h. **(B)** Effect of CT on cytokine expression levels. Total RNA was collected from A549 cells after exposure to a series of concentrations of CT for 24 h. RT-PCR was performed with corresponding primers. One-way ANOVA with Dunnett’s post-test was used to analyze the data for statistical differences, *n* = 3, *p < 0.05, **p < 0.01, ***p < 0.001 compared with the control group. **(C)** Effect of CT on the transcription of GSDMs. Cluster analysis of GSDMs gene expression in A549 cells treated with 10, 15 or 20 μM CT for 24 h.

Previous studies reveal that CT is a pro-inflammatory metabolite that triggers an inflammatory response in various types of cells (Liu et al., 1997; Liao et al., 2009; Voisin et al., 2017; Kloudova-Spalenkova et al., 2020; Fernandes et al., 2024). To investigate the role of CT in inflammatory responses, we firstly performed RNA-seq to evaluate the differential expression of inflammation-related genes in CT-treated A549 cells. CT treatment identified 273 differentially expressed genes (DEGs) related to the immune system (fold change ±1.5). KEGG pathway enrichment analysis revealed that the most significantly enriched pathway was the NLRP signaling pathway (Supplementary Figure S1A). Enrichment of IL-17, cytokine pathways, and necrotic apoptosis pathways suggests that CT may regulate inflammation by inducing inflammatory cell death. By clustering the inflammation-related genes, we found that the transcription of pro-inflammatory cytokines (IL-6, IL-15, and IL-23) and chemokine-related genes (CCL20, CXCL1, CXCL2, CXCL3, and CXCL5) were increased (Figure 1A). Besides, the increased expression levels of IL-6, IL-15, and IL-18 were observed following CT treatment in an RT-PCR assay (Figure 1B). Conversely, IL-1β, IL-10, IL-12, and IL-23 expression showed no significant changes (Supplementary Figure S1B). Meanwhile, we also analyzed the expression of the inflammatory death effector proteins. RNA-seq also indicated that GSDME had the highest expression among inflammatory death effector proteins in A549 cells (Figure 1C).

Several NLRP family members form inflammasomes closely linked to pyroptosis (Platnich and Muruve, 2019; Yu et al., 2020). However, we detected no expression of NLRP3, which is one of the most common NLRP family members (Supplementary Figure S1C). Meanwhile, CT did not increase the expression of precursor IL-1β (pro-IL-1β) and pro-IL-18 proteins in A549 cells (Supplementary Figure S1C). These results demonstrate that CT promotes inflammatory cell death through alternative pathways.

Flow cytometry was employed to identify the cytotoxic effects of CT on cancer cells. CT increased the proportion of annexin V^+^/PI^+^ cells in a dose- and time-dependent manner (Supplementary Figure S2A,B). Our previous study has demonstrated that CT induces both apoptosis and lytic cell death characterized by membrane disruption (Chen et al., 2024). CT is associated with inflammatory response, which are downstream effector molecules of inflammatory cell death (e.g., GSDM family proteins) known to induce plasma membrane perforation (Liu et al., 2021; Yu et al., 2021). Thus, CT possibly regulates inflammatory responses by inducing inflammatory cell death.

### CT induces GSDME-mediated pyroptosis

**FIGURE 2:**
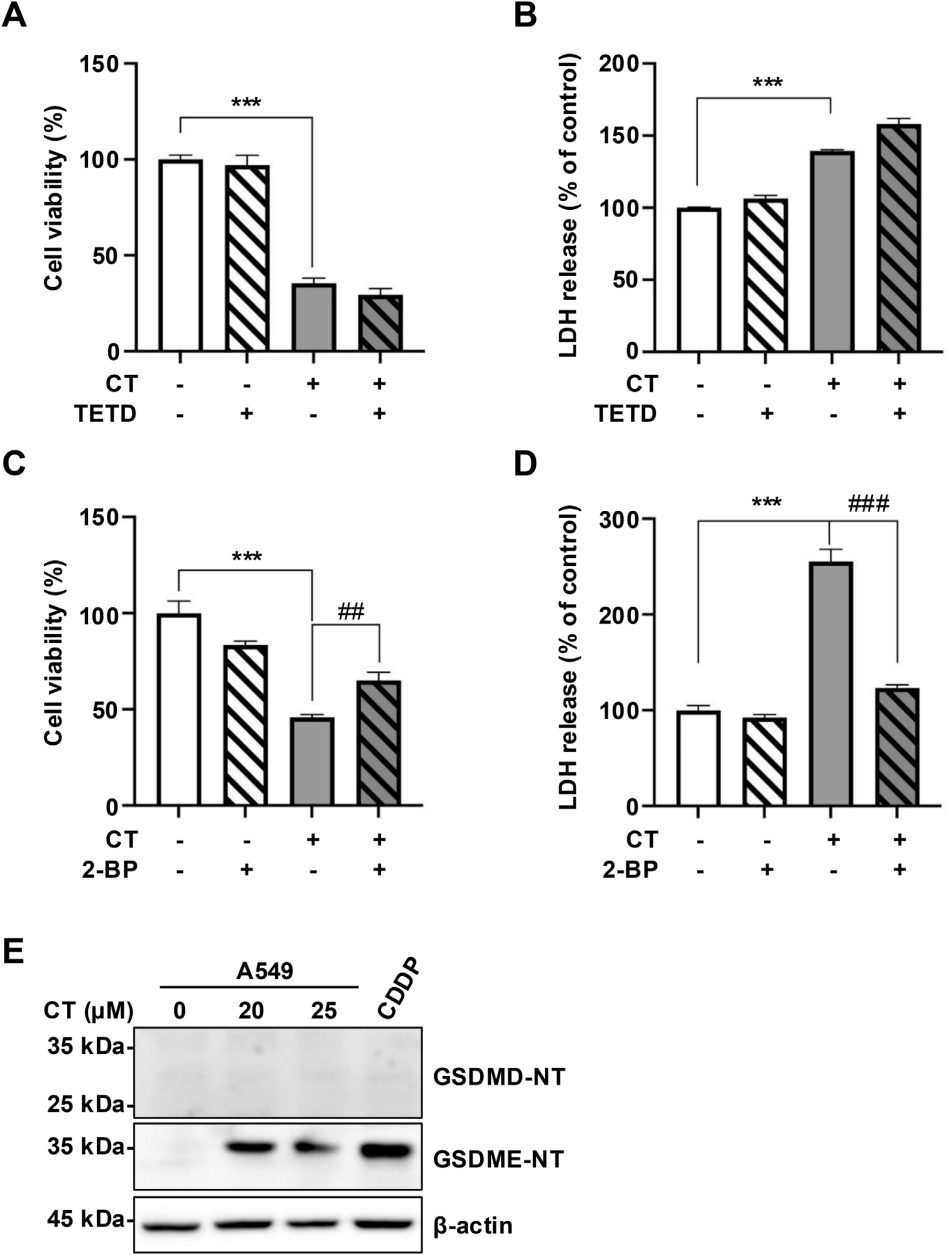
CT induced GSDME activation in A549 cells. **(A–D)** The effect of pyroptosis inhibitors on CT-induced cell death in cell viability or LDH released assays. Cells were pre-treated with 10 µM TETD or 10 µM 2-BP for 1 h, then incubated with 20 µM CT for 24 h. Statistical significance was determined using one-way ANOVA with Tukey’s post-test, *n* = 3, ***p < 0.001 compared with NC group, ##*p* < 0.01, ###*p* < 0.001 compared with CT group. **(E)** The levels of GSDMs N-terminal in cells treated with CT. The cells were treated with 20 or 25 µM CT for 24 h. Cells treated with 40 µM CDDP for 24 h were the positive control group.

Given that CT treatment induced GSDME expression, we investigated whether CT triggers GSDME-mediated pyroptosis using pathway-specific inhibitors and pyroptosis markers. With the exception of PJVK, all GSDM family members can form membrane pores through their N-terminal domains (Liu et al., 2021; Yu et al., 2021). We examined the roles of two key members, GSDMD and GSDME, using pharmacological inhibitors, TETD, which covalently modifies Cys191 in human GSDMD or Cys192 in mouse GSDMD to block pore formation (Hu J. J. et al., 2020), and 2-BP, which inhibits GSDME palmitoylation by binding palmitoyltransferases, thereby preventing N-terminal domain of GSDME (GSDME-NT) release and pore formation (Hu L. et al., 2020). In CT-treated A549 cells, co-treatment with 2-BP significantly attenuated cytotoxicity (assessed by cell viability and LDH release assays), whereas TETD conferred no protection (Figures 2A–D). We further analyzed the cleavage of GSDME-NT and GSDMD-NT. CDDP served as a positive control for caspase 3/GSDME-mediated pyroptosis (Zhang et al., 2019). Immunoblotting revealed no detectable GSDMD-NT, while CT treatment substantially increased GSDME-NT levels (Figure 2E), indicating GSDME-specific activation.

We excluded other forms of cell death induced by CT, including necroptosis and ferroptosis. Nec-1s, a recognized necroptosis inhibitor (Chen et al., 2024), did not alleviate the cell death or LDH release caused by CT (Supplementary Figure S2C,D). Meanwhile, WB results showed MLKL phosphorylation in the positive group (TSZ treated-HT29 cells), but not in CT treated-A549 cells (Supplementary Figure S2E), indicating CT does not induce necroptosis in A549 cells. Similarly, a CCK-8 assay was performed to evaluate the effects of ferroptosis inhibitors on CT-induced cell death. DFP inhibits ferroptosis by directly chelating with iron ions and reducing excess iron ions, while Lip-1 and Fer-1 prevent ferroptosis by suppressing lipid peroxidation (Du and Guo, 2022). As illustrated in Supplementary Figure S2F, these ferroptosis inhibitors were ineffective in preventing cell death induced by CT. Ferroptosis is usually accompanied by lipid peroxidation (Feng and Stockwell, 2018). Ferroptosis detection typically uses BODIPY-C11 to assess membrane lipid peroxide levels (Martinez et al., 2020). Our results showed that CT increased the level of lipid ROS dose-dependently (Supplementary Figure S2G), which was consistent with reported CT-induced accumulation of secondary products of lipid peroxidation in cells (Wu and Huang, 2006; Liu et al., 2011). Therefore, CT cannot induce ferroptosis in A549 cells.

Collectively, these findings suggest that CT-induced GSDME activation, rather than GSDMD, plays a critical role in inflammatory cell death, thereby regulating inflammatory responses.

### GSDME-mediated pyroptosis is the main form of programmed cell death induced by CT

To determine whether pyroptosis is the primary type of cell death triggered by CT-treatment, we examined morphological characteristics of CT-treated cells (Zhang et al., 2019). Microscopic analysis revealed characteristic pyroptotic membrane bubbling (large bubbles) (Supplementary Figure S3A), resulting from cell membrane perforation and osmotic imbalance. These morphological findings indicate that CT induces pyroptosis in A549 cells.

**FIGURE 3:**
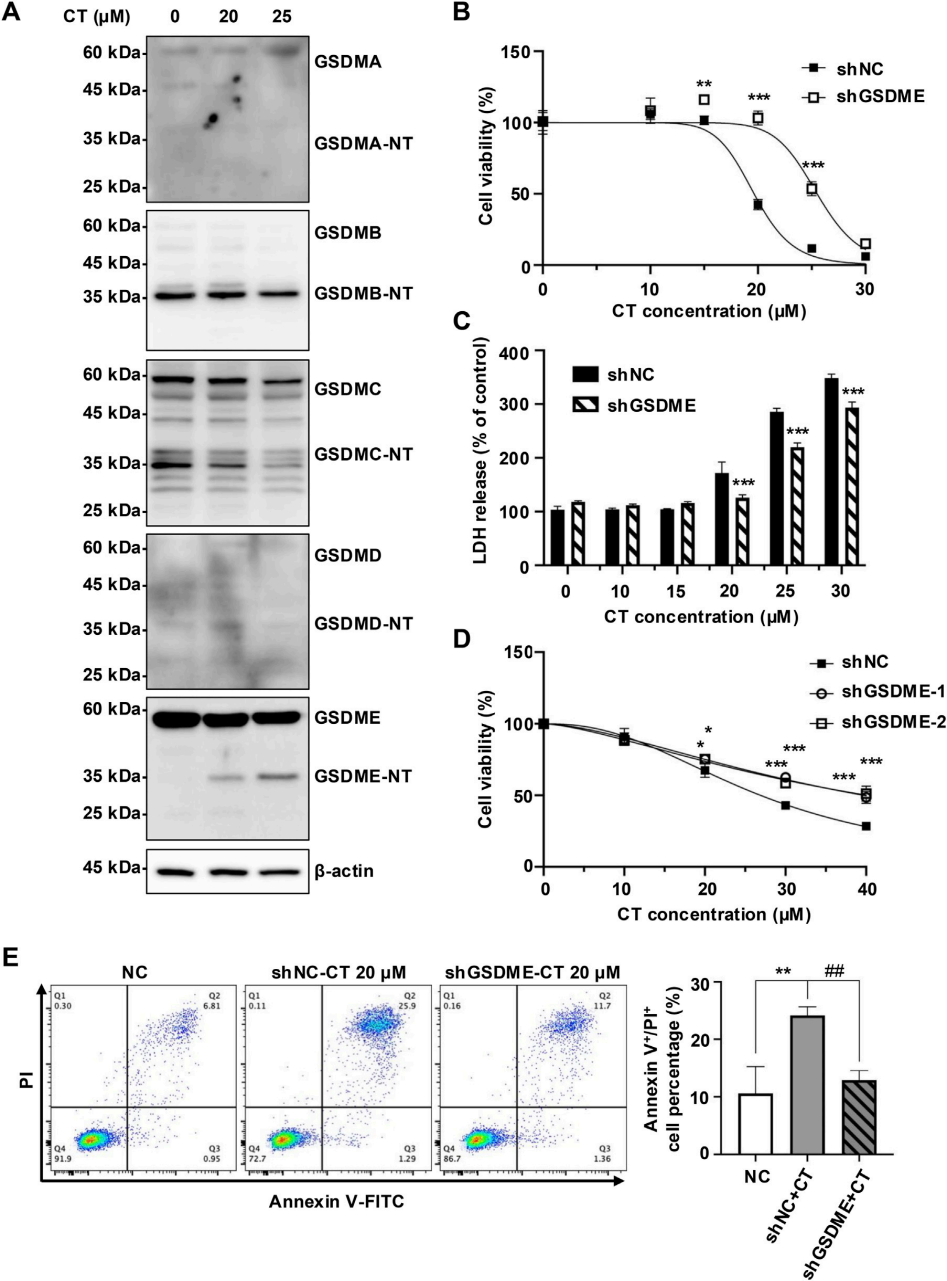
CT promoted early cell death by specifically activating GSDME. **(A)** The effect of CT on the activation of GSDMs in A549 cells. After the cells were treated with 20 or 25 μM CT for 24 h, the cell lysate was collected and quantified for protein expression detection. **(B)** Knockdown of GSDME inhibited CT-induced A549 cell death. The cells were treated with a series of concentrations of CT for 24 h. The data were analyzed by two-way ANOVA with Šídák’s post-test, *n* = 3, **p < 0.01, ***p < 0.001, compared with the shNC group at the same dose of CT treatment. **(C)** The effect of GSDME knockdown on CT-induced LDH release in A549 cells. Two-way ANOVA with Šídák’s post-test was used to analyze the data, *n* = 3, ***p < 0.001 compared with the shNC group at the same dose of CT treatment. shGSDME-2 (shGSDME). **(D)** The effect of silencing GSDME on CT-decreased HeLa cell survival. After shGSDME-HeLa cells were treated with a series of doses of CT for 24 h, the CCK-8 assay was performed. Two-way ANOVA with Dunnett’s post-test was used to analyze the data for statistical differences, *n* = 3, *p < 0.05, ***p < 0.001 compared with the shNC group. **(E)** Annexin V/PI staining of shGSDME-A549 cells after 24 h of treatment with 20 µM CT. The left images were the representative results of each group, and the right was the statistical graph of all sample results. Statistical significance was determined using one-way ANOVA with Tukey’s post-test, *n* = 3, **p < 0.01 compared with NC group, ##*p* < 0.01 compared with CT group.

To further assess the role of GSDME in CT-induced pyroptosis, we analyzed cleavage of GSDM proteins in CT-treated A549 cells. Immunoblotting demonstrated decreased full-length GSDME (55 kDa), accompanied by an increase in GSDME-NT (30–35 kDa) in CT-treated A549 cells, while other GSDM members were not affected (Figure 3A). These results suggest that GSDME is the specific pyroptosis effector in this process. Notably, CT selectively activated GSDME across multiple cell lines despite GSDMD presence (Supplementary Figure S3B).

To investigate GSDME dependence, we generated GSDME-silenced A549 cells (Supplementary Figure S3C). Knockdown of GSDME significantly reduced cellular sensitivity of A549 cells to CT-induced death (Figures 3B,C). Moreover, shGSDME-A549 cells maintained a higher survival rate than the negative control (NC) group at CT doses ≥25 µM (Figures 3B,C). This GSDME dependence was further validated in GSDME-silenced HeLa cells (Figure 3D; Supplementary Figure S3D). Reduced GSDME expression also attenuated CT-induced membrane damage, evidenced by decreased Annexin V/PI double-positive cells (Figure 3E). Collectively, these results indicate that GSDME-mediated pyroptosis is a critical mechanism in CT-induced cytotoxicity, particularly during early death phases.

### Caspase 3 is essential for CT-induced pyroptosis

**FIGURE 4:**
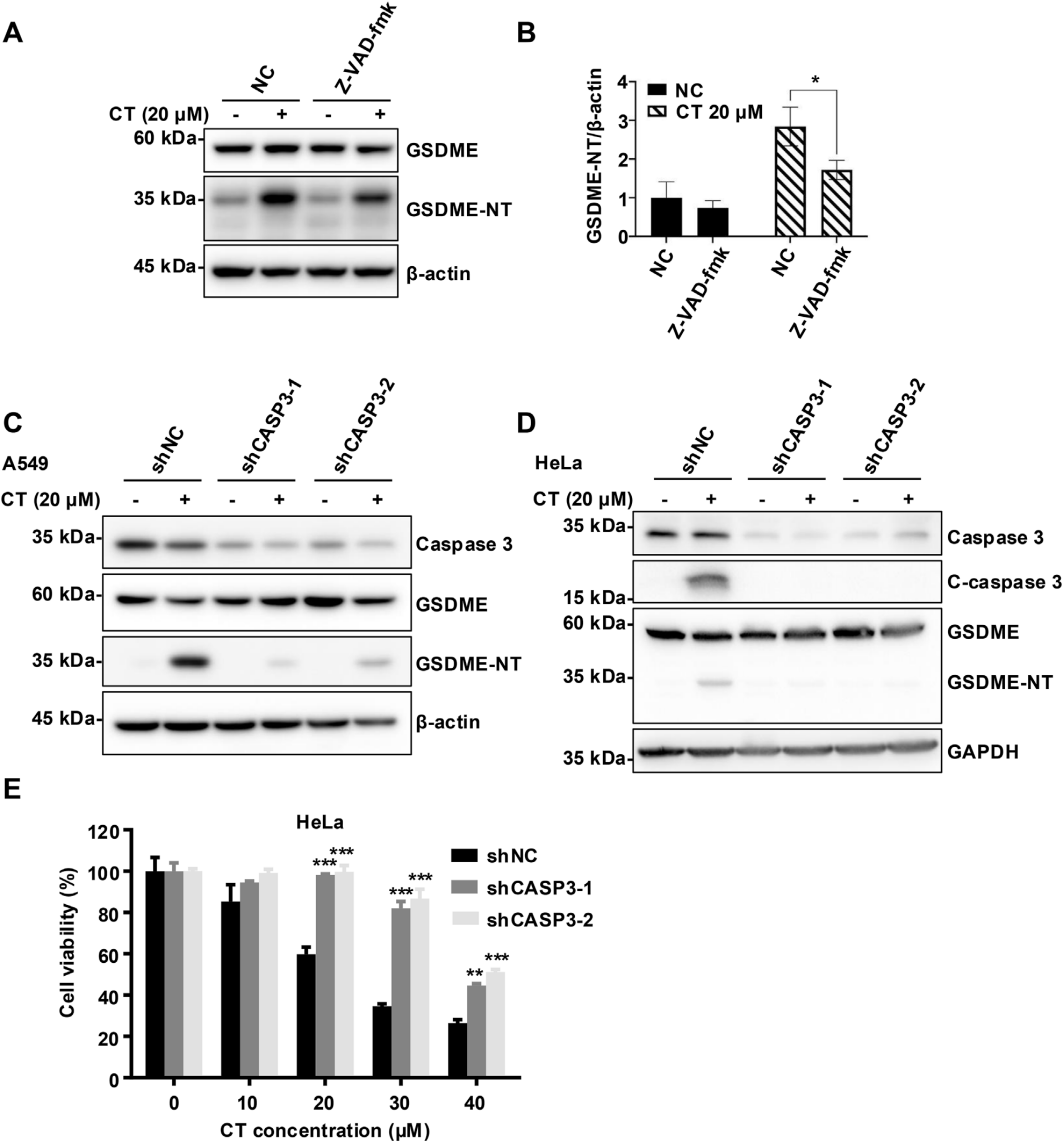
CT activated GSDME in a caspase 3-dependent manner. **(A)** The effect of caspase 3 inhibitor Z-VAD-fmk on CT-induced GSDME activation and **(B)** quantification of GSDME-NT levels in A549 cells. After 20 µM Z-VAD-fmk pre-treatment for 1 h, cells were co-treated with 20 µM CT for 24 h. Cells were lysed and quantified for protein expression. Data are represented as mean ± SD of three different experiments. Statistical significance was determined using two-way ANOVA with Šídák’s post-test, *n* = 3. *p < 0.05 compared with the NC group. The effect of silencing caspase 3 on CT-induced GSDME activation in **(C)** A549 cells and **(D)** HeLa cells. After shCASP3-A549 or shCSAP3-HeLa cells were treated with 20 µM CT for 24 h, cells were lysed and quantified for protein expression. **(E)** The effect of silencing caspase 3 on CT-decreased cell survival. After shCASP3-HeLa cells were treated with a series of doses of CT for 24 h, the CCK-8 assay was performed. Two-way ANOVA with Dunnett’s post-test was used to analyze the data for statistical differences, *n* = 3, ***p < 0.001 compared with the NC group.

We then investigated the molecular mechanism of CT-induced pyroptosis. The prolonged exposure in WB analysis was necessary to visualize the band of c-caspase 3, indicating a slight activation of caspase 3 in CT-treated A549 cells (Chen et al., 2024). Consistent with this, CT treatment activated and cleaved caspase 3 in HeLa cells (Figure 4D). Besides, the pan-caspase inhibitor Z-VAD-fmk suppressed the activation of GSDME but failed to completely abolish it, potentially reflecting incomplete caspase-3 inhibition during prolonged CT exposure (Figures 4A,B; Supplementary Figure S4A,B). To further validate the role of caspase 3 in this process, we employed RNA interference to specifically silence caspase 3 expression. Notably, silencing caspase 3 effectively prevented GSDME activation (Figures 4C,D) and CT-induced cell death (Figure 4E), indicating caspase 3 is crucial for the cytotoxicity of CT. Collectively, these findings imply that c-caspase 3 is the upstream protease that promotes the cleavage of the N-terminal of GSDME.

## Discussion

Oxysterols are known to associate with numerous major pathologies through modulation of various crucial cellular processes (Zmyslowski and Szterk, 2019; de Freitas et al., 2021; Samadi et al., 2021). CT is one of the most abundant auto-oxidative cholesterol in mammals (Helmschrodt et al., 2013).

While some reports have proposed the possibility that CT mediates the inflammatory responses, the precise mechanisms remain undefined (Liu et al., 1997; Liao et al., 2009). In this study, RNA-seq analysis revealed CT potently induces multiple inflammatory factors. The role of this effect in chronic inflammatory diseases remains to be further investigated.

*In vitro* research has indicated that CT exhibits potency in promoting apoptosis (Liu et al., 2004; Liu et al., 2005; Carvalho et al., 2010). However, our findings have demonstrated that the priority cell death induced by CT extends beyond apoptosis (Chen et al., 2024), suggesting involvement of additional mechanisms. GSDME participates in the pathogenesis of inflammatory diseases by triggering pyroptosis, leading to the release of proinflammatory cytokines (Tan et al., 2021). At the same time, through transcriptomics analysis and protein level detection, it was found that only GSDME was significantly expressed in A549. Through morphological assessment and selective cell death inhibitors screening, we confirmed that CT is capable of inducing pyroptosis. GSDME silencing effectively attenuated CT cytotoxicity, however, this protection diminished at higher CT concentrations (Figures 3B–D). When both apoptosis and pyroptosis were occurring in cells, pyroptosis occurred more rapidly than apoptosis (Wang et al., 2017), potentially elucidating the failure of GSDME knockdown to mitigate cell death at elevated CT concentrations. This assumption was consistent with our previous study that CT predominantly triggers GSDME-mediated pyroptosis, accompanied by a minor degree of apoptosis (Chen et al., 2024). Importantly, we observed that the specific activation of GSDME by CT may be a common phenomenon in multiple cell lines (Supplementary Figure S3B).

Caspase 3 is currently recognized as the sole upstream activator of GSDME, cleaving GSDME protein at Asp270 to generate a necrotic N-terminal fragment. This fragment interacts with the plasma membrane (PM) to initiate pyroptosis (Rogers et al., 2017; Bhat et al., 2023). Furthermore, GSDME appears to amplify the activation of caspase 3, thereby forming a positive feedback loop (Jiang et al., 2020; Liao et al., 2022). Although Z-VAD-fmk only partially impeded the cleavage of GSDME, silencing caspase 3 robustly suppressed GSDME activation in both A549 and HeLa cell lines (Figures 4C,D). Consequently, caspase 3 is an essential driver of CT-induced GSDME activation.

This study revealed a novel form of cell death induced by CT through activation of caspase 3/GSDME to trigger pyroptosis. However, it is puzzling that CT does not activate canonical inflammatory signaling pathways, such as IL-1β and NLRP3, despite inducing inflammatory response and cytokine genes (Supplementary Figure S1A–C). Thus, how CT induces shearing of GSDME through activation of caspase 3 remains a mystery to be explored.

**FIGURE 5:**
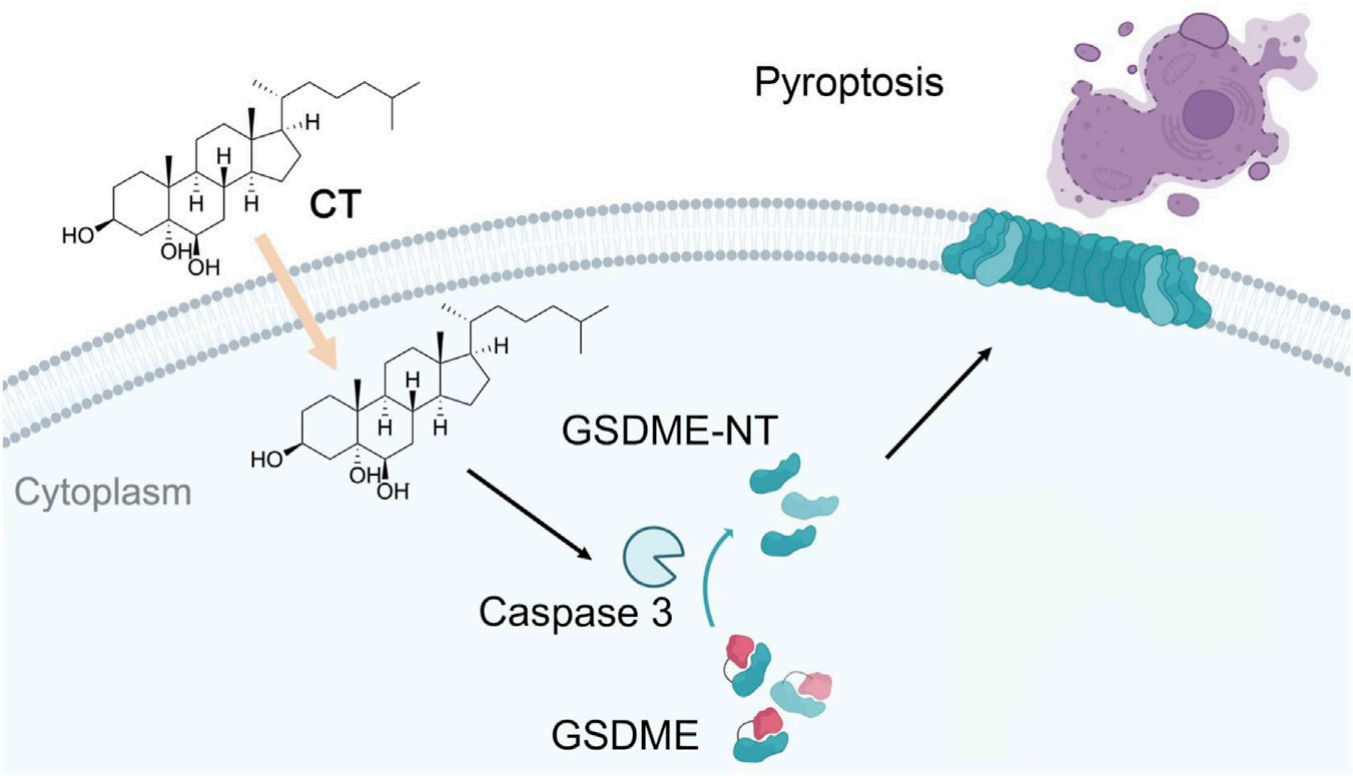
The mechanism of CT induces pyroptosis through a GSDME-mediated pathway. CT specifically activates GSDME cleavage in a caspase 3-dependent manner and subsequently leads to pyroptosis.

Another autoxidized cholesterol further supported the role of oxysterols in cell death. 7-KC similarly induces both necrosis and GSDME-mediated pyroptosis in retinal pigment epithelium cells (Pariente et al., 2023). The current study also described that CT exacerbates inflammation by promoting the GSDME-mediated pyroptosis process (Figure 5). This discovery provides compelling evidence that autoxidized cholesterols regulate inflammation via programmed cell death and inflammatory response process. Therefore, investigating the association between pyroptosis and CT may provide valuable insights into the role of CT in inflammation. The research on the relationship between autoxidized cholesterols and inflammation will offer novel therapeutic targets for related diseases.

## Data Availability

The original contributions presented in the study are publicly available. This data can be found here: https://ngdc.cncb.ac.cn/gsa-human, HRA013764.

## Glossarry

2-BP: 2-bromohexadecanoic acid
7-KC: 7-ketocholesterol
Akt: protein kinase B
c-caspase 3: cleaved caspase 3
CCK-8: Cell Counting Kit-8
CDDP: cisplatin
COX-2: cyclooxygenase-2
CT: cholestane-3β,5α,6β-triol
DEGs: differentially expressed genes
DFP: deferiprone
eNOS: endothelial nitric oxide synthase
Fer-1: ferrostatin-1
GSDMs: gasdermins
GSDME-N-T: N-terminal domain of GSDME
IL-8: interleukin 8
LDH: lactate dehydrogenase
Lip-1: liproxstatin-1
MLKL: mixed lineage kinase domain-like pseudokinase
NC: negative control
Nec-1s: Necrostatin-1 stable
NLRP3: NOD-like receptor protein 3
PI3K: phosphoinositide 3-kinase
PJVK: Pejvakin
PM: plasma membrane
pro-IL-1β: precursor IL-1β
RNA-seq: RNA-sequencing
ROS: reactive oxygen species
RT-PCR: Real-time PCR
STS: staurosporine
TETD: disulfiram
WB: western blot.
