# Supplementary material for: Cholestane-3β,5α,6β-triol induces cancer cell death by activating GSDME-mediated pyroptosis
Source: Front Pharmacol. 2025 Oct 24;16:1667156. doi: 10.3389/fphar.2025.1667156 (PMC12592123; doi:10.3389/fphar.2025.1667156)
Supplement: Supplementary file 1 [file Supplementaryfile1.docx]

Supplementary Material


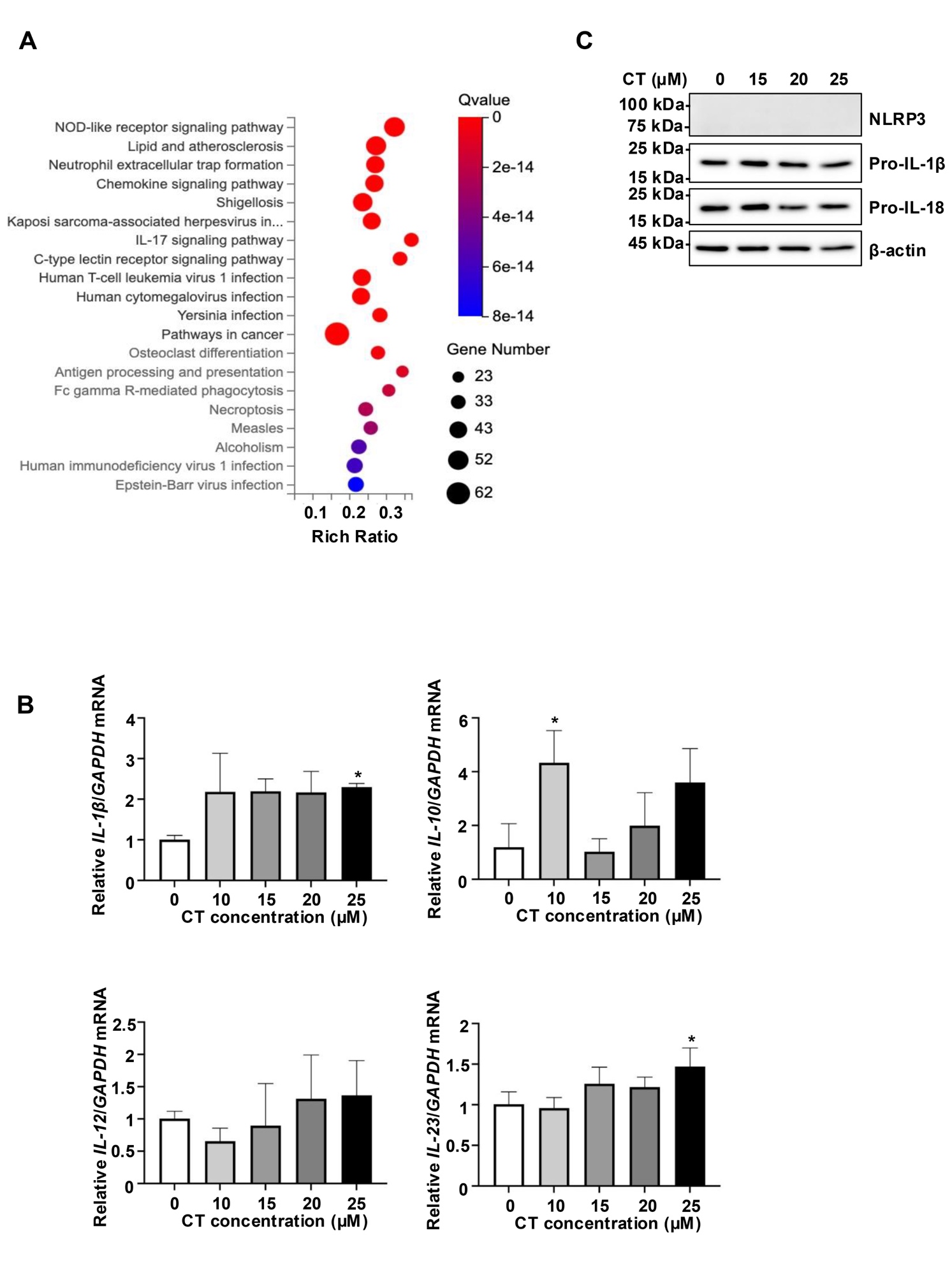


**Supplementary Figure S1.** **Effect of CT on the expression levels of cytokines in A549 cells. (A)** The enrichment of the KEGG pathway in A549 cells treated with CT. Cluster analysis of different expression genes (fold change ± 1.5) related to the immune system in A549 cells treated with 20 µM CT for 24 h. **(B)** Effect of CT on cytokine expression levels. Total RNA was collected from A549 cells after exposure to different concentrations of CT for 24 h. RT-PCR was performed with the corresponding primers. One-way ANOVA with Dunnett’s post-test was used to analyze the data for statistical differences, *n* = 3, * *p* < 0.05 compared with the control group. **(C)** Expression levels of NLRP3 and related cytokine precursors. A549 cells were treated with a series of concentrations of CT for 24 h, and cell lysates were collected and quantified for WB. Pro-IL-1β, precursor IL-1β; Pro-IL-18, precursor IL-18.


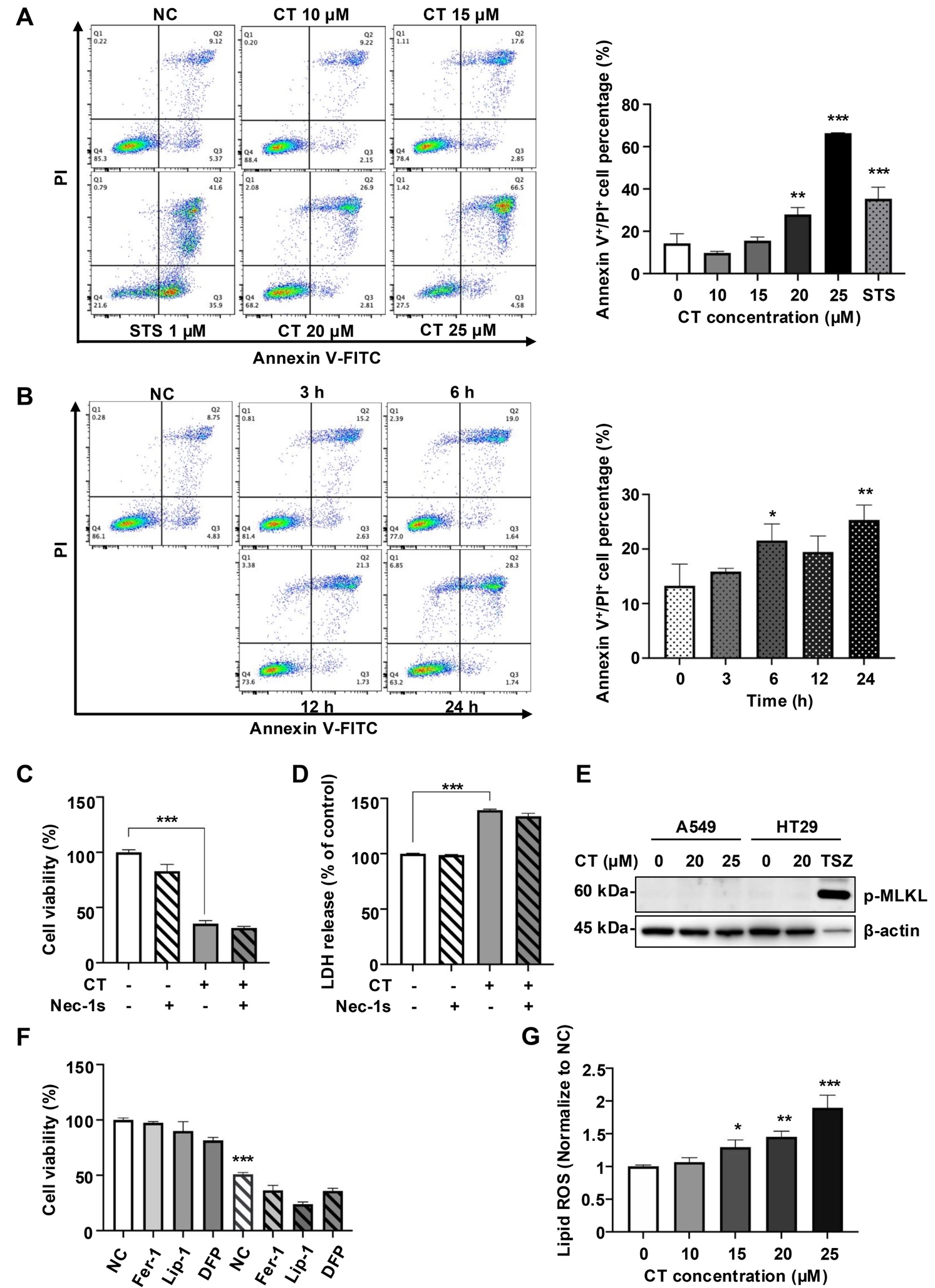


**Supplementary Figure S2.** **CT dose- and time-dependently induced A549 cell death. (A)** A549 cells were treated with different concentrations of CT for 24 h and subjected to annexin V and PI double staining. Cells treated with staurosporine (STS) 1 µM for 24 h were used as the positive control. The data statistics were shown in the right bar chart. **(B)** A549 cells were treated with 20 µM CT for 3, 6, 12, and 24 h and subjected to annexin V and PI double staining. The data statistics were shown in the right bar chart. Statistical significance was determined using one-way ANOVA with Dunnett’s post-test, * *p* < 0.05, ** *p* < 0.01, *** *p* < 0.001 compared with the negative control (NC) group. **(C)** Cells were pre-treated with 10 µM Nec-1s for 1 h, then incubated with 20 µM CT for 24 h. Cell viability and **(D)** LDH release were measured. Statistical significance was determined using one-way ANOVA with Tukey’s post-test, *n* = 3, *** *p* < 0.001 compared with the NC group. **(E)** The level of p-MLKL in A549 and HT29 cell lines treated with CT for 24 h or TSZ for 4 h, respectively. **(F)** The effect of ferroptosis inhibitors on CT-induced cell death. Cells were pre-treated with 5 µM Fer-1, 5 µM Lip-1, or 20 µM DFP for 1 h, then incubated with 20 µM CT for 24 h. Cell viability was assessed using a CCK-8 kit. Statistical significance was determined using one-way ANOVA with Tukey’s post-test, *n* = 3. *** *p* < 0.001 compared with the NC group. **(G)** The levels of cellular lipid ROS were detected by the BODIPY-C11 probe. A549 cells were treated with a series of doses of CT for 24 h. Statistical significance was determined using one-way ANOVA with Dunnett’s post-test, * *p* < 0.05, ** *p* < 0.01, *** *p* < 0.001 compared with the NC group.

**
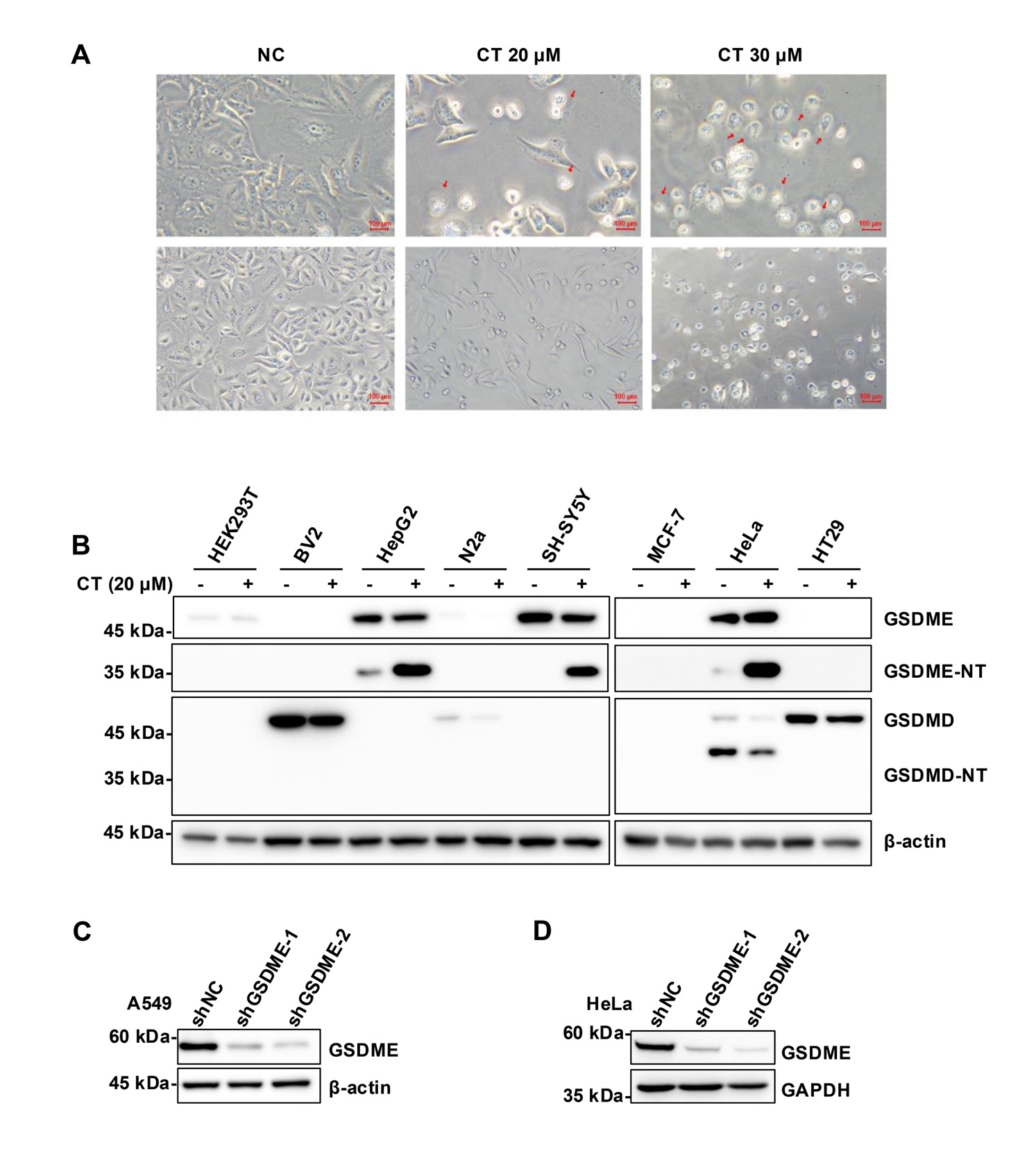
**

**Supplementary Figure S3.** **CT-induced pyroptosis was dependent on the cleavage of GSDME. (A)** Representative images of CT-induced pyroptosis in A549 cells. Bright-field images of A549 cells were collected after being treated with 20 µM or 30 µM of CT for 24 h. The upper and lower rows of images were taken under a microscope at different magnifications (scale bar: 100 μm). The left column was the control group, the middle column was the 20 μM CT treatment group, and the right column was the 30 μM CT treatment group. The red arrow showed the large bubbles formed on the PM during pyroptosis. **(B)** The activation of GSDME and GSDMD in different cell lines under 20 µM CT treatment. After treating cells with 20 µM CT for 24 h, cell lysates were collected and quantified. (**C**) The establishment of shGSDME-A549 cell lines and **(D)** shGSDME-HeLa cell lines. After being screened by 0.5 µg/mL puromycin for more than 3 days, cells expressing shGSDME sequences were lysed and quantified for protein expression detection.

**
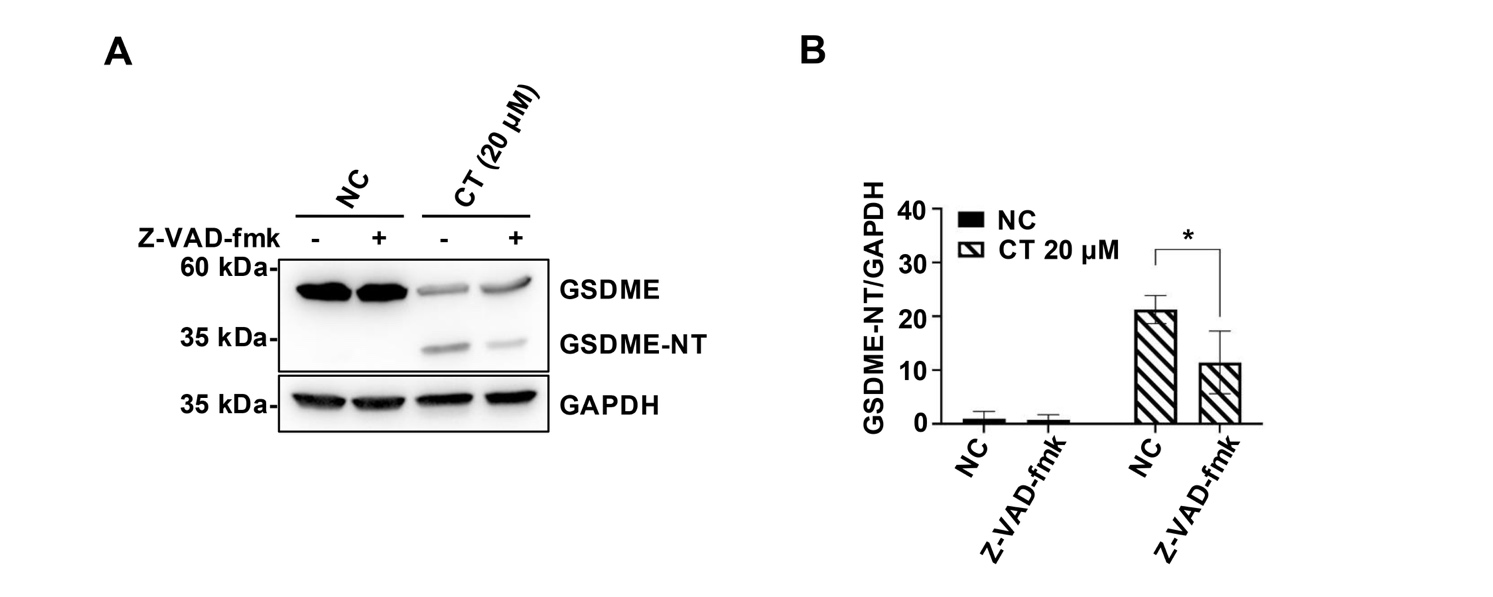
**

**Supplementary Figure S4.** **CT activated GSDME in a caspase 3-dependent manner. (A)** The effect of caspase 3 inhibitor Z-VAD-fmk on CT-induced GSDME activation and **(B)** quantiﬁcation of GSDME-NT levels in HeLa cells. After 20 µM Z-VAD-fmk pre-treatment for 1 h, cells were co-treated with 20 µM CT for 24 h. Cells were lysed and quantified for protein expression. Data are represented as mean ± SD of three diﬀerent experiments. Statistical significance was determined using two-way ANOVA with Šídák's post-test, *n* = 3. * *p* < 0.05 compared with the NC group.

**Table S1.** **The sequence of shRNA.**

| **Plasmids** | **Sequence (5'-3')** |
| --- | --- |
| shGSDME-1 | GCATGATGAATGACCTGACTT |
| shGSDME-2 | GATGATGGAGTATCTGATCTT |
| shCASP3-1 | CCGAAAGGTGGCAACAGAATT |
| shCASP3-2 | GTGGAATTGATGCGTGATGTT |
